# Testicular volumes revisited: A proposal for a simple clinical method that can closely match the volumes obtained by ultrasound and its clinical application

**DOI:** 10.1186/1687-9856-2012-17

**Published:** 2012-06-08

**Authors:** Juan F Sotos, Naomi J Tokar

**Affiliations:** 1 Department of Pediatric, College of Medicine, The Ohio State University, Nationwide Children’s Hospital, Section of Pediatric Endocrinology, Metabolism & Diabetes, 700 Children’s Drive, Columbus, OH, 43205, USA; 2 Nationwide Children’s Hospital, Section of Pediatric Endocrinology, Metabolism & Diabetes, 700 Children’s Drive, Columbus, OH, 43205, USA

**Keywords:** Testicular volume, Gonadal development, Pubertal changes

## Abstract

**Background:**

The testicular volumes obtained with the clinical methods, calculated using the ellipsoid equation W^2^ x L x π/6, correlate with those obtained by ultrasound (US) and are useful clinically, but overestimate ultrasound values, mainly because of the inclusion of the scrotal skin and epididymis, have much variability, and may not be accurate or reproducible.

The US measurement is somewhat inconvenient, because it requires another procedure and, mainly, is costly.

It would be helpful to have a simple, low cost clinical method that approximates or closely matches the results obtained by ultrasound.

Formulas, equivalent to the ellipsoid equations, were developed to calculate testicular volumes with corrections of the width (W), length (L), and height (H) of the testis obtained in the scrotum to avoid the inclusion of the scrotal skin and epididymis.

**Subjects & methods:**

The US observations in our hospital of the width, height, length, height/width, and length/width ratios and volumes of 110 testes from 55 children from 1 month to 17 ½ years of age were reviewed. Based on these observations and those reported by others, formulas to apply to the clinical measurements were developed to approximate the volumes obtained by ultrasound. The validity and accuracy of the formulas were determined. For the clinical application of the formulas, measurements of the width of the testis in the scrotum, with a centimeter ruler, were obtained in 187 study subjects in different stages of puberty and adults, for a total of 374 testicular determinations.

**Results:**

The widths obtained in the scrotum were corrected by subtracting the values of the double scrotal skin. The formulas were then applied and the testicular volumes determined. The testicular volumes were then compared to the ultrasound values reported in hundreds of subjects by four different groups and statistically analyzed. The volumes obtained by the formulas (means ± SD) closely matched the volumes obtained by ultrasound.

**Conclusion:**

A simple clinical method, based on the width of the testis obtained in the scrotum with a centimeter ruler, which can determine testicular volumes closely matching those reported by ultrasound, is proposed.

## Background

For more than 50 years there has been [1-4] and continues to be [5-7] an interest in the subject. The determination of the testicular volume is of considerable importance to assess for a number of conditions: the onset, progression and disorders of puberty, the effect of cryptorchidism and orchiopexy, hypogonadism with respect to tubular function, the effect of a varicocele, abnormal testicular development, damage to the testis by torsion or inflammation, compensatory hypertrophy, detection of Klinefelter syndrome, effect of the administration of sexual steroids or drugs, and, in adults, assessment of fertility. Testicular size correlates with tubular size, function and spermatogenesis [8].

In addition, the testicular volume is of interest to assess macroorchidism, such as in Fragile X syndrome, FSH secreting pituitary macroadenomas, long-standing hypothyroidism, adrenal rest cell tumors in congenital adrenal hyperplasia, lymphomas and so on.

A number of clinical methods have been used for the measurement of testicular volumes in the scrotum. Some use the length and width or the testis obtained with an ordinary ruler or with sliding calipers [2,3]. Others use orchidometers by comparative palpation with ellipsoid models of known volume [1,9,10] or by a series of punch out elliptical rings of varying sizes that fit over the testis [11,12]. All the clinical methods calculate the volumes following the ellipsoid equation W^2^ x L x 0.52.

Ultrasound measurements of testicular volume have a high degree of accuracy and reproducibility and are the standard for quantitation of testicular volume [13-15]. The volumes obtained with the clinical methods correlate with those obtained by ultrasound and are useful clinically, but overestimate ultrasound values [14-17], by two to three folds [7,16], mainly because the inclusion of the scrotal skin and epididymis, have much intraobserver and interobserver variability and may not be accurate or reproducible [13]. The ultrasound measurement, however, is somewhat inconvenient, because it requires another procedure, and, mainly, is costly. It does not appear practical or reasonable to use ultrasound to assess the onset and progression of puberty or to assess some of the other conditions that have been mentioned.

It would be helpful to have a clinical method that is simple, low cost, and that approximates or closely matches the results obtained by ultrasound.

The volumes obtained by ultrasound have been calculated by different ellipsoid equations. Some have used only the width (W) and length (L) of the testes, W^2^ x L x π/6 that when resolved is W^2^x L x 0.52 = Volume. More frequently they have included the height (H), W x H x L x 0.52 and others, recently, have used the constant 0.71 to closely match the “true” testicular volumes obtained by water displacement, W x H x L x 0.71 = Volume.

Three formulas, equivalent to the 3 ellipsoid equations used in ultrasound measurements, were developed with corrections of the width, length, and height of the testis obtained in the scrotum, to avoid the inclusion of the scrotal skin and epididymis and to approximate testicular volumes obtained by ultrasound.

The aim of this report is to describe a simple clinical method based on the width of the testis obtained in the scrotum with a centimeter ruler that can determine testicular volumes closely matching those reported by ultrasound. 1) The basis for the development of the formulas to do so, 2) their validity and accuracy, and 3) the volumes obtained with the formulas in our children, adolescents and adults will be presented.

## Subjects and methods

The ultrasound observations in our hospital, of the width, height, length, height/width and length/width ratios, and volumes of 110 testes from 55 children, from 1 month to 17 ½ years of age (using Phillips, Model iu22 and Siemens S2000 with linear array transducers and imaging frequencies of 17-5 MHz and 18-16 MHz respectively) were reviewed.

Based on these observations and those reported by others, formulas were developed to approximate the volumes obtained by ultrasound, by expressing the width (W), without the scrotal skin (W-ss) and the height (H) (anterior-posterior diameter or depth) and length (L) in the ellipsoid equation W x H x L x 0.52 with values based on the ratios of the Width-ss; the height as a ratio of the width (H/(W-ss)), to avoid the inclusion of the scrotal skin and body of the epididymis, and the length as a ratio of the width (L/(W-ss)) to avoid the inclusion of the head of the epididymis and scrotal skin. Three formulas were developed to be equivalent to the ellipsoid equations used in US measurements: to the equation W^2^ x L x 0.52 (Formula 1); to the equation W x H x L x 0.52 (Formula 2); and to the equation W x H x L x 0.71 (Formula 3).

The validity and accuracy of the formulas were determined by the significance of Pearson’s linear correlations coefficient (r) and by the comparison of the volumes obtained by ultrasound and by the formulas.

For the clinical application of the formulas, 163 measurements of the double scrotal skin (ss) were obtained with a Harpenden Skinfold caliper in boys in different stages of puberty and in adults. Measurements of the width and length of the testis in the scrotum, with a centimeter ruler, were obtained in 187 study subjects in different stages of puberty and in adults, for a total of 374 testicular determinations. The 187 subjects consisted of 42 normal and 145 patients attending the endocrine clinic who had normal growth and gonadal development. The widths obtained in the scrotum were corrected by subtracting the values for the double scrotal skin, in accordance with their gonadal stage, to approximate or match the width of the testis. The formulas were then applied and the testicular volumes determined. The testicular volumes obtained were then compared to the ultrasound volumes reported by four different groups.

To avoid confusion, the term “equation” has been used for the determination of testicular volumes by ultrasound and “formula” for the calculation of volumes in study subjects.

### Basis for the development of the formulas

The testis is assumed and generally accepted to be an ellipsoid. When the width and the height are the same (a prolate spheroid, like a rugby ball), the equation for the volume would be: W^2^ x L x ^π^/_6_ = volume or W^2^ x L x 0.52 = volume – that comes for the resolution of the equation: (^W^/_2_)^2^ x π x ^4^/_3_ x ^L^/_2_ = volume. If the width and the height are different, as in a rotational ellipsoid, then the equation would be W x H x L x 0.52.

As previously mentioned, all the clinical measurements of the testes overestimate the US volumes, mainly because of the inclusion of the scrotal skin and the epididymis.

Formulas, to apply to the clinical measurements obtained in the scrotum of the study subjects, were developed to approximate the volumes obtained by US, by expressing the width (W) without the scrotal skin (W-ss), the height (H) as a ratio of the width (H/(W-ss)) and the length and a ratio of the width (L/(W-ss)). The numbers for the last two ratios can be obtained by the measurements usually observed in ultrasound determinations.

#### A. Length/width ratio

With the development and growth of the testes, the dimensions of the testes width and length remain proportional with the width being approximately 2/3 of the length [9,11]. The width/length ratio in a number of testes (number 110) in our hospital by US was 0.64 ± 0.09 (length/width ratio = 1.55 ± 0.21). The width/length ratio determined by ultrasound in a number of children by others was 0.67 ± 0.12 (length/width ratio = 1.5) [13]. Of note is that the width/length ratio of all the ellipsoid models from 1 ml to 25 ml in the Prader orchidometer is the same, 0.638 (length/width = 1.57), and of all the punch out elliptical rings in the Takihara (also known as the Rochester Orchidometer) is 0.666 (length/width = 1.50) [12]. Thus, knowing the width, the length of the testis can be calculated (L = (W-ss) x 1.55 or 1.50) and the length would not include the epididymis and the scrotal skin. So the length in the formula can be expressed as 1.55 x (W-ss) = L.

#### B. Height/width ratio

The testis is not a perfect prolate spheroid, but an ellipsoid. The height or depth is usually less than the width by ultrasound measurements. As a consequence the US volumes obtained by the equations W^2^ x L x 0.52 or W x H x L x 0.52 are different. The volumes are lower with the equation that includes the height.

By report of US measurements, it seems that the height is variable. In our hospital the H/W ratio in 110 testes was 0.76 ± 0.12 (minimum 0.5 to maximum 1.0). The H/W ratio of 0.69 ± 0.04 (minimum 0.69 to maximum 0.8) was reported by Osemlak [6]. Thus, the H/W ratio to be included in the formula may need to be adjusted in different institutions. With our data the best correlation was obtained with an H/W ratio of 0.8.

Thus, the H in the formula could be expressed as 0.8 x (W-ss) = H. Because of the variability of the shape in the testes (or in the US measurements), there would be a variability of the results. Higher values of the H/W ratio of the testes by US than in the formula will yield higher values by US than the formula and vice versa.

#### C. Formulas

Thus, Formula 1, equivalent to the ellipsoid equation W^2^ x L x 0.52, would be (W-ss)^2^ x ((W-ss) x 1.55) x 0.52 = volume or (W-ss)^3^ x 0.80.

Formula 2, equivalent to the ellipsoid equation W x H x L x 0.52, would be (W-ss) x (0.8 x (W-ss)) x (1.55 x (W-ss)) x 0.52 = volume or (W-ss)^3^ x 0.64.

Formula 3, equivalent to the ellipsoid equation W x H x L x 0.71 would be the same as formula 2, except for the constant of 0.71 instead of 0.52, (W-ss) x (0.8 x (W-ss)) x (1.55 x (W-ss)) x 0.71 = volume or (W-ss)^3^ x 0.88.

The equation W x H x L x 0.71 = volume comes from the observations of Lambert [18]. In postmortem dissections, Lambert found that the constant of 0.52 results in too small of a value for testicular size and concluded that the constant to be used in practice should be 0.71. The values of the constant, however, varied from 0.37 to 1.08, depending on the shape and size of the testis. Consequently, he recognized that the error (or variability) of the method was quite large. This variability relates to the different shape of the testis, and would be difficult to resolve.

Ultrasound measurement of the testicular volume is acknowledged to be the best method to quantitate the size of the testis. There is some question regarding the equation that should be used to obtain the volume of the testis.

The equation W x H x L x 0.52 = volume, is probably the most frequently used for ultrasound measurements. According to some authors that equation underestimates the “true” volumes determined by water displacement and the equation W x H x L x 0.71 is the best and should be used [19,20].

Thus, with the measurement of the width of the testis in the scrotum, and subtraction of the double scrotal skin one could determine the volume.

Some variability related to the variability of the shape of the testis itself and intra and interobserver variability would be expected.

The formula or ellipsoid equation used should be the same for clinical and for US methods.

If no height is included: W^2^ x L x 0.52 = volume – for the ultrasound,

(1) $${\left(W-ss\right)}^{3}\times 0.80=\mathrm{volume}for\;the\;clinical\;method\text{.}$$

If height is included: W x H x L x 0.52 = volume for the ultrasound,

(2) $${\left(W-ss\right)}^{3}\times 0.64=\mathrm{volume}for\;the\;clinical\;method\text{.}$$

If the constant of 0.71 instead of 0.52 is used: W x H x L x 0.71 = volume for the ultrasound,

(3) $${\left(W-ss\right)}^{3}\times 0.88=\mathrm{volume}for\;the\;clinical\;method\text{.}$$

The constant will change, depending on the H/W ratio included in the formula, which should be based on the H/W ratio in the institution.

## Statistics

The correlations between the volumes obtained by ultrasound and by the formulas were measured using the Pearson’s correlation coefficients (r). The significance of the difference between the means of the samples was calculated by paired *t*-test. All tests were two-tailed and significance was set at p <0.05.

## Results

### Validity and accuracy of the formulas

The validity and accuracy of the formulas were determined by the significance of the linear correlations and by the comparison of the volumes obtained by ultrasound and by the formulas.

The ultrasound results of 110 testicular measurements (width, height, length) and volumes obtained in our hospital were used.

To assess the validity of the formulas, the same widths were used for the ultrasound and for the formulas.

#### Formula1

**Figure 1:**
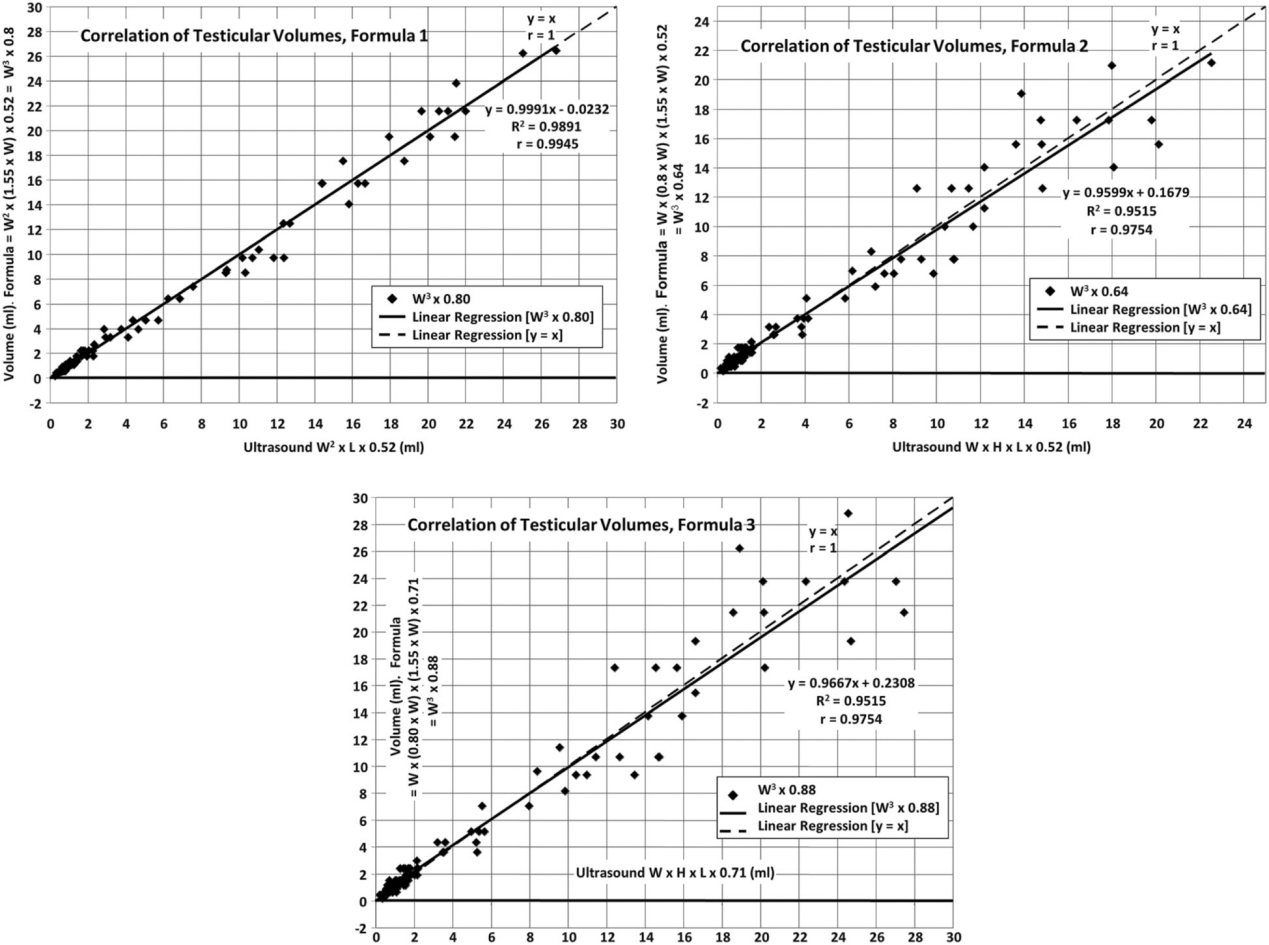
a, b, c [Correlations of Testicular Volumes, 3 charts] “Correlations of testicular volumes obtained by US and volumes calculated by the formulas”.

The correlation of the testicular volumes obtained by ultrasound, without the inclusion of height (W^2^ x L x 0.52 = volume) and the formula (W^2^ x (1.55 x W) x 0.52) W^3^ x 0.8 = volume is illustrated in Figure 1a. In 110 determinations, the correlation coefficient was r = 0.9945, highly significant (p <0.001) and the regression equation y = 0.9991x – 0.0232. The mean of the volumes of 5.27 ± 6.90 ml by US and 5.24 ± 6.93 ml by formula were not different (p = >0.5)(not shown). Because when one has large numbers and differences in gonadal development the means may not reflect the large variation, the volumes by age groups were compared (Table 1). Again, the volumes were not different (p >0.5).

**Table 1:** Comparison of Testicular Volumes Obtained by Ultrasound (US) in our Hospital with those obtained by Formulas (means ± SD) All using the US Width

**Age range** **years**	**Number**	**US (ml) equation** **W^2^ x L x 0.52**	**Formula (ml)** **W^3^ X 0.8**	**p value**
1 to 2	19	0.64 ± 0.25	0.64 ± 0.24	>0.5
3 to 6	26	1.05 ± 0.44	1.08 ± 0.50	>0.5
7 to 9	15	1.37 ± 0.56	1.46 ± 0.63	>0.5
10 to 11	9	1.40 ± 0.60	1.57 ± 0.77	>0.5
12 to 13	18	7.79 ± 4.40	7.83 ± 4.81	>0.5
14 to 15	10	12.87 ± 7.12	12.50 ± 7.43	>0.5
16 to 17	13	18.31 ± 4.63	18.02 ± 5.54	>0.5
**Age range ** **years**	**Number**	**US (ml) equation** **W x H x L x 0.52**	**Formula(ml)** **W**^**3**^**x 0.64**	**p value**
1 to 2	19	0.48 ± 0.17	0.51 ± 0.20	>0.5
3 to 6	26	0.78 ± 0.34	0.87 ± 0.40	>0.1
7 to 9	15	0.95 ± 0.42	1.17 ± 0.51	>0.1
10 to 11	9	1.06 ± 0.35	1.26 ± 0.61	>0.1
12 to 13	18	5.94 ± 3.31	6.26 ± 3.85	>0.5
14 to 15	10	10.02 ± 4.76	9.99 ± 5.95	>0.5
16 to 17	13	15.49 ± 4.06	14.42 ± 4.44	>0.5
**Age range** **years**	**Number**	**US (ml) equation** **W x H x L x 0.71**	**Formula(ml)** **W**^**3**^**x 0.88**	**p value**
1 to 2	19	0.65 ± 0.24	0.70 ± 0.27	>0.5
3 to 6	26	1.06 ± 0.46	1.19 ± 0.55	>0.1
7 to 9	15	1.30 ± 0.58	1.61 ± 0.70	>0.1
10 to 11	9	1.45 ± 0.48	1.73 ± 0.84	>0.1
12 to 13	18	8.10 ± 4.52	8.61 ± 5.21	>0.5
14 to 15	10	13.68 ± 6.50	13.75 ± 8.18	>0.5
16 to 17	13	21.15 ± 5.54	19.83 ± 6.10	>0.5

#### Formula 2

The correlation of the testicular volumes obtained by ultrasound in 110 testes, when the height was included (W x H x L x 0.52), and by the formula (W x (0.8 x W) x (1.55 x W) x 0.52) W^3^ x 0.64 = volume, is shown in Figure 1b.

The correlation coefficient was r = 0.97545, highly significant (p <0.001), and the regression equation Y = 0.9599x +0.1679.

The mean of the volumes by US and the formula were not different (p >0.5), 4.19 ± 5.63 ml and 4.19 ± 5.54 ml (not shown). Again the volumes were compared by age groups. The volumes were not different (p >0.1 or >0.5) (Table 1).

#### Formula 3

Because of reports [19,20] that using the constant 0.71, instead of 0.52, is the best to determine the true volume of the testis, obtained by water displacement, calculations and comparisons were made using the 0.71 constant.

The correlation of the testicular volumes obtained by US (W x H x L x 0.71 = volume) and by the formula (W x (0.8 x W) x (1.55 x W) x 0.71) W^3^ x 0.88 is shown in Figure 1c.

The correlation coefficient was r = 0.9754, highly significant (p <0.001), and the regression equation y = 0.9667x + 0.2308.

The means of the volumes by US and by the formula were 5.73 ± 7.69 ml and 5.77 ± 7.62 ml, respectively, not different (not shown). Again the volumes were compared by age groups. The volumes were not different (p >0.1 or >0.5) (Table 1).

By the aforementioned, the formulas seem valid and accurate. The variability of results is owing to the variability of the shape of the testis, variability on the length/width ratio or height/width ratio. This variability is difficult to resolve, because the US measurements are individual and the method applies the same formula for all.

### Clinical application of the formulas to our study subjects

Measurements of the double scrotal skin were obtained (Table 2), so that the width of the testis obtained in the scrotum could be corrected to approximate or equal the width of the testis.

**Table 2:** Double Scrotal Skin (cm)

**Gonadal stage**	**Number of measurements**	**Mean ± SD**
G-1	36	0.17 ± 0.02
G-2	28	0.15 ± 0.02
G-3	18	0.16 ± 0.01
G-4	31	0.19 ± 0.02
G-5	22	0.20 ± 0.03
Adults	28	0.21 ± 0.03

**Legend:** Measurement obtained within 1 to 2 seconds after releasing the grip using a Harpenden Skinfold Caliper HSK-BI.

The double scrotal skin measured 0.17 cm for G-1, 0.15 for G-2, 0.15 for G-3, 0.19 for G-4, 0.2 for G-5 and 0.21 for adults.

The width and length of the testis in the scrotum were measured, with a centimeter ruler, in 187 boys in different stages of puberty and in adults, for a total of 374 testes. Our study subjects were divided in groups by age (to permit comparison with other published reports) or by Tanner stages of pubertal development.

With the widths obtained after subtracting the double scrotal skin, the formulas were applied to our study subjects and the volumes were compared to the testicular volumes obtained by ultrasound by others: to P Osemlak [6] who reported volumes in 309 boys from 1 day to 17 years of age (linear array transducer 12 MHz LA523); to J Goede et al. [7] who obtained volumes in 769 boys 6 months to 19 years of age (using a 12 MHz linear array transducer – Falco AutoImage, Falco Software, Tomsk, Russia); to Kuijper et al. [21] who reported volumes in the first 6 years of life in 344 boys obtained with a linear array transducer 7.5 MHz (Aloka SSD-900); and to JY Bahk et al. [22] who determined volumes in 1,139 normal young men, 19 -27 year old by ultrasound (model SSD, 1700 Aloka, Japan) (Table 3).

**Table 3:** Testicular Volumes (ml) Obtained by Ultrasound in Normal Children and Adults Reported by 4 Groups Compared with Volumes in Our Study Subjects Obtained Clinically by Formula

**Age group**	**Osemlak**[6] **Mean ± SD**	**Goede et al.**[7] **Mean ± SD**	**Kuijper et al.**[21] **Mean ± SD**		**Our study subjects** (W-ss)^3^ x 0.64 **Mean ± SD**
1 month	(17) 0.35 ± 0.12		(31) 0.27 ± 0.02		
2 to 12 months	(17) 0.5 ± 0.24	(40) 0.48 ± 0.13	(216) 0.44 ± 0.03 0.31 ± 0.02		
2 years	(17) 0.55 ± 0.22	(38) 0.46 ± 0.09	0.31		
3 years	(17) 0.64 ± 0.19	(36) 0.51 ± 0.15	0.31		(24) 0.46 ± 0.07
4 years	(17) 0.78 ± 0.21	(38) 0.51 ± 0.16	0.31		
5 years	(17) 0.67 ± 0.19	(48) 0.58 ± 0.15	0.31		
6 years	(17) 0.78 ± 0.24	(42) 0.63 ± 0.26	0.31		
7 years	(17) 0.68 ± 0.21	(62) 0.65 ± 0.17			(22) 0.56 ± 0.09
8 years	(17) 0.81 ± 0.23	(59) 0.66 ± 0.22			
9 years	(17) 0.85 ± 0.31	(53) 0.79 ± 0.46			(36) 0.65 ± 0.19
10 years	(18) 1.36 ± 0.61	(49) 0.97 ± 0.51			
11 years	(18) 1.94 ± 1.41	(60) 1.33 ± 1.03			(50) 2.56 ± 1.24
12 years	(17) 3.29 ± 2.99	(55) 2.33 ± 1.77			
13 years	(18) 5.37 ± 2.92	(47) 4.42 ± 2.66			(18) 4.28 ± 0.96
14 years	(17) 4.98 ± 2.68	(35) 7.31 ± 4.11			(58) 8.01 ± 2.58
15 years	(17) 8.71 ± 2.52	(26) 8.69 ± 2.91			
16 years	(17) 11.8 ± 4.91	(31) 11.51 ± 3.03			(36) 12.45 ± 1.99
17 years	(17) 12.83 ± 3.94	(27) 12.12 ± 2.8			
18 years		(23) 13.73 ± 3.51			(56) 13.16 ± 2.67
Adults	**Bahk et al.**[22] W x H x L x 0.52				(W-ss)^3^ x 0.64
	(1139)	*Lt. 13.46 ± 2.65 Rt. 13.29 ± 2.82			(102) 13.12 ± 3.17
	W x H x L x 0.71				(W-ss)^3^ x 0.88
	(1139)	Lt. 18.37 ± 3.62 Rt. 18.13 ± 3.85			(102) 18.05 ± 4.36

**Legend:** (#) Number of observations. The US equation used by Osemlak and Goede et al. and Kuijper et al. was W x H x L x 0.52.

* The equation used by Bahk et al. was W x H x L x 0.71. The volumes were divided by 1.365 to obtain volumes determined with the constant 0.52 (0.71/0.52 = 1.365).

For the first nine years, all the volumes, on the average, are less than 1 ml and the means of our children are on the range reported by others.

Our values seem lower than those reported by Osemlak and Goede et al. by 0.1 to 0.2 ml for the first 9 years of age. Although this difference is statistically significant (p < 0.05), it does not appear of clinical importance. This difference could result from a 0.5 to 1 mm difference in the measurement of the width, by manual compression. Kuijper et al. reported an increase of the volumes from 0.27 ml at 1 month to 0.44 at five months (minipuberty), and a decrease to 0.31 ml at 9 months. The volumes remain stable after that. They did not report the number of observations or standard deviations of the 97 children 1 to 6 years of age, so no comparison could be analyzed.

The volumes after 10 years seem similar, even though the age of some groups was not the same. Statistical comparisons showed no differences (p >0.1 or >0.5) (Table 4).

**Table 4:** Comparison of Testicular Volumes (ml) Obtained by Ultrasound in Normal Children and Adults at different ages Reported by 3 Groups with Volumes in Our Study Subjects Obtained Clinically (means ± SD)

**Age group**	**Osemlak**[6]	**Goede et al.**[7]	**Bahk et al.**[22] **(W x H x L x 0.52)**	**Our study subjects** **(W-ss)^3^ x 0.64**	**p value** **Osemlak to** ** Our**	**p value ** **Goede et al. ** **To Our**	**p value** **Bahk et al. ** **To Our**
5 years	(17) 0.67 ± 0.19	(48) 0.58 ± 0.15		(24) 0.46 ± 0.07	<0.001	<0.001	
7 years	(17) 0.68 ± 0.21	(62) 0.65 ± 0.17		(22) 0.56 ± 0.09	<0.05	<0.01	
9 years	(17) 0.85 ± 0.31	(53) 0.79 ± 0.46		(36) 0.65 ± 0.19	<0.02	>0.05	
12 years	(17) 3.29 ± 2.99	(55) 2.33 ± 1.77		(50) 2.56 ± 1.24	>0.1	>0.05	
13 years	(18) 5.37 ± 2.92	(47) 4.42 ± 2.66		(18) 4.28 ± 0.96	>0.1	>0.05	
15 years	(17) 8.71 ± 2.52	(26) 8.69 ± 2.91		(58) 8.01 ± 2.58	>0.1	>0.1	
17 years	(17) 12.83 ± 3.94	(27) 12.12 ± 2.8		(36) 12.45 ± 1.99	>0.5	>0.5	
18 years		(23) 13.73 ± 3.51		(56) 13.16 ± 2.67		>0.1	
Adults			(1139)*13.29 ± 2.82	(102) 13.12 ± 3.17			>0.1
			(W x H x L x 0.71) 18.37 ± 3.62	((W-ss)^3^ x 0.88) (102) 18.05 ± 4.36			>0.1

**Legend:** (#) Number of observations.

The equation used by Osemlak and Goede et al. was W x H x L x 0.52 and our formula (W-ss)^3^ x 0.64.

* Calculated from the values using 0.71 as a constant.

**Figure 2:**
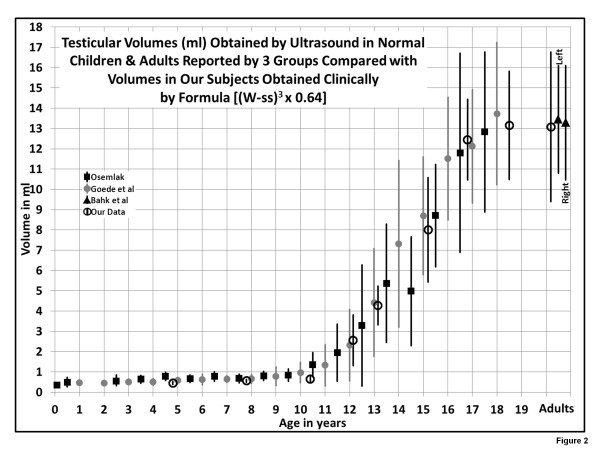
**[Comparison of Testicular Volumes obtained by the formula & those published by 3 groups, chart] “Illustration of the means ± SD of volumes obtained by US by Osemlak**[6]**, Goede et al. **[7]**, Bahk et al. **[22]**, and our formula.** The formula (W-ss)^3^ x 0.64 is equivalent to the US equation W x H x L x 0.52”.

Figure 2 showing the means and standard deviations of the different groups is rather convincing that the volumes of our study subjects, based on the formula, closely match the volumes obtained by ultrasound in different institutions. This, in itself, is additional evidence in support of the validity and accuracy of the formulas.

**Figure 3:**
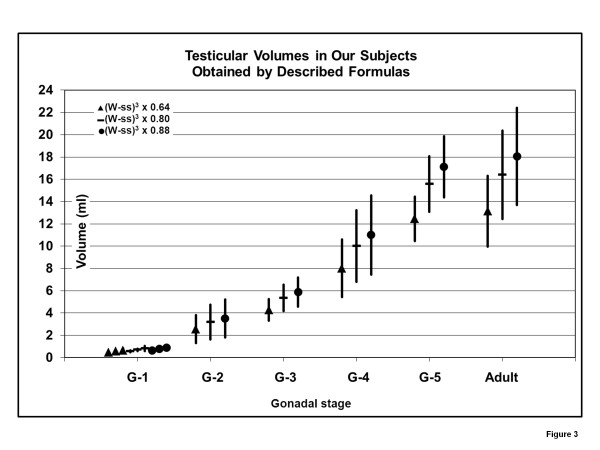
**[Testicular Volumes at gonadal stages with 3 formulas, chart] “Illustration of the means ± SD of volumes at different stages of gonadal (G) development.** The formula with the constant 0.64 is equivalent to the equation W x H x L x 0.52; the one with the constant 0.80 is equivalent to W^2^ x L x 0.52; the one with the constant 0.88 is equivalent to W x H x L x 0.71”.

Because of the wide range of ages for the development of gonadal stages and the overlapping of ages for different stages (i.e. G-1 up to 13 9/12 years; G-2, 9 to 13; G-3, 12 to 16), it seems preferable to report the volumes obtained in study subjects with the formulas by the gonadal stage (Table 5 and Figure 3). Pubertal stages were determined by the method of Tanner.

**Table 5:** Testicular Volumes (ml) of Study Subjects Obtained Clinically by Described Formulas

	**Formulas equivalent to the following US Equations**		
**Gonadal Stage**	**W**^**2**^**x L x 0.52**	**W x H x L x 0.52**	**W x H x L x 0.71**
**(number in group)**	**(W-ss)**^**3**^**x 0.8** **(mean ± SD)**	**(W-ss)**^**3**^**x 0.64** **(mean ± SD)**	**(W-ss)**^**3**^**x 0.88** **(mean ± SD)**
G-1 3 to 7 yr (24)	0.57 ± 0.09	0.46 ± 0.07	0.63 ± 0.10
7 to 9 yr (22)	0.70 ± 0.11	0.56 ± 0.09	0.77 ± 0.12
9 to 11 yr (36)	0.81 ± 0.24	0.65 ± 0.19	0.90 ± 0.26
G-2 (50)	3.20 ± 1.56	2.56 ± 1.24	3.52 ± 1.71
G-3 (18)	5.36 ± 1.20	4.28 ± 0.96	5.89 ± 1.32
G-4 (58)	10.01 ± 3.22	8.01 ± 2.58	11.01 ± 3.55
G-5 (36)	15.57 ± 2.49	12.45 ± 1.99	17.12 ± 2.74
Adults (102)	16.41 ± 3.96	13.12 ± 3.17	18.05 ± 4.36

One can convert the volumes and standard deviations from one formula to another by multiplying or dividing

(4) $$\begin{array}{l} \mathrm{From}{\left(W-ss\right)}^{3}\times 0.8\mathrm{to}{\left(W-ss\right)}^{3}\times 0.88\text{,}\text{multiply}\text{by}\;1.1\;\left(\text{since}0.88/0.8=1.1\right)\\ \text{From}{\left(W-ss\right)}^{3}\times 0.8\text{to}{\left(W-ss\right)}^{3}\times 0.64\left(0.8/0.64=1.25\right)\text{divide}\text{by}\;1.25\text{or}\text{vice}\;\text{versa}\text{.} \end{array}$$

If one prefers the formula with the constant 0.71 to obtain the volumes that approximate “true” testicular volumes, then multiply (W-ss)^3^ x 0.64 by 1.365, since 0.88/0.6448 = 1.365].

## Discussion

The measurement of the testicular volume is not an exact science. After the advent in 1970, ultrasound has been recognized as the most accurate and reproducible method, even though variability related to the transducer used, possibility of compression of the testis, and intra and interobserver variation in the measurements (width, height, length, and volumes), among other factors exists [13,19,21].

Different methods have been used for the clinical measurement of testicular volumes: measurements of the testis in the scrotum by a ruler or by a caliper or by orchidometers. A number of orchidometers have been described: the Prader orchidometer, described in 1966 [9], and the Takihara (also known as the Rochester orchidometer), described in 1983 [12], are probably the most frequently used.

There have been multiple publications comparing the volumes obtained by the orchidometers and by ultrasound. The volumes obtained with the clinical methods correlate with those obtained by ultrasound and are useful clinically, but all overestimate the volumes obtained by ultrasound, have much variability and may not be accurate or reproducible.

A simple clinical method that would approximate or closely match the ultrasound values would be quite helpful.

In 1966, Prader stated [9] that “knowing the width of the testis in the scrotum (obtained by a caliper), one can calculate the volume, being assumed that the testicle is an ellipsoid of revolution, corresponding to the equation 0.52 x W^2^ x L or 0.71 x W^2^ x L”. In the ellipsoid the width is about 2/3 of the length. Since he felt that the use of the caliper was laborious and required considerable manipulation, he developed ellipsoid models of known volumes for comparison, all of them with an L/W ratio of 1.57 (W/L = 0.638), and volumes calculated using the equation 0.52 x W^2^ x L.

The ultrasound method was not available then. The volumes obtained with the Prader orchidometer overestimate those obtained by ultrasound [7,13,14,16,19,20] usually by 2 to 3 folds, because of the inclusion of the scrotal skin and epididymis, the lack of including the height of the testis, and the intraobserver and, particularly, the interobserver variability.

The method presented here, more or less, states the same as was stated in 1966. Knowing the width of the testis in the scrotum (with a centimeter ruler), one can calculate the volume, but this time closely matching the ultrasound values. To do that, the width was subtracted by the double scrotal skin to approximate the width obtained by US, the length was expressed as a ratio of the width to avoid the inclusion of the epididymis and scrotal skin, and the height was expressed as a ratio of the width, to take into consideration the inclusion of the height in the ultrasound measurements and to avoid the inclusion of the scrotal skin and the body of the epididymis.

Thus, formulas were developed to be equivalent to ultrasound equations (W^2^ x L x 0.52, W x H x L x 0.52, or W x H x L x 0.71).

The validity and accuracy of the formulas were determined by the significance of the linear correlations and by the comparison of the volumes obtained by ultrasound and by the formulas.

The formulas were applied to the clinical measurements obtained in 374 testes in our study subjects and the volumes compared to the volumes obtained by ultrasound by 4 different groups.

The results seem rather convincing that the testicular volumes of our study subjects, based on the formulas, closely match the volumes obtained by ultrasound in different institutions.

The proposed method should be helpful to assess the onset and progression and disorders of puberty and the disorders previously mentioned.

The US remains the method of choice for the evaluation of extratesticular (i.e. hydrocele, spermatocele, epididymal cyst, varicocele) or intratesticular (i.e. tumors) abnormalities.

As always, there may be limitations. The clinical measurements were obtained by one observer. The interobserver variability remains to be determined.

Ultrasound determinations could be obtained in the same subjects whom the clinical measurements are made and then compare US volumes with those obtained by formulas. Comparison with US volumes reported by others, as done, would seem to be a more difficult test, so different results may not be likely.

In summary: A simple clinical method, based on the width of the testis obtained in the scrotum with a centimeter ruler that can determine testicular volumes closely matching those reported by ultrasound is proposed. This method should be helpful for the assessment of the onset and progression of puberty, of disorders of puberty and of conditions associated with differential testicular volumes. (Appendix)

A centimeter ruler is usually available to any provider and should be less intrusive than the use of a caliper or orchidometer.

The process for the determination of the testicular volume seems simple:

 1. Measurement of the width of testis in the scrotum can be obtained by smoothing the scrotal skin around the testis with the thumb and index finger of one hand, avoiding compression of the testis and using the ruler with the other hand.

 2. The Tanner Stage of pubertal (gonadal) development is determined.

 3. The width is subtracted by the double scrotal skin, for the gonadal stage – shown in Table 2. One could make it simpler by subtracting 1.5 mm for Tanner stages 1, 2, and 3 and 2 mm for Tanner 4, 5, and adult. The error or variation would be minor.

 4. The volume, then, is calculated by the formula: (W-ss)^3^ x 0.88, if one would like to obtain the “true” volume of the testis matching volumes determine by water displacement, or by (W-ss)^3^ x 0.64 or (W-ss)^3^ x 0.8 and compared with the normal values for the Tanner (gonadal) stage and adults shown in Tables 5 and Figure 3.

 5. If one would like to compare the values obtained by the formula with those obtained by ultrasound in the institution, one should use the formula equivalent to the ellipsoid equation that they use for the calculation of US volumes: for US equation W^2^ x L x 0.52 use formula (W-ss)^3^ x 0.8; for US equation W x H x L x 0.52 use formula (W-ss)^3^ x 0.64; and for US equation W x H x L x 0.71 use formula (W-ss)^3^ x 0.88.

## Appendix

### Assessment of differential testicular volumes

The formulas can also provide information on the testicular volumes expected from the changes in millimeters of the width (Table 6) and be helpful for evaluation of disorders associated with discrepancies in testicular volumes.

Of particular interest is the effect of a varicocele, occurring in approximately 10 to 25% of adolescents and adults, more commonly (85 to 95%) in the left scrotum. The varicocele may lead to testicular asymmetry from an arrest of growth of the testis in adolescents and to testicular atrophy in adults, thought to result from apoptosis of Sertoli cells owing to increased temperature from blood. Small size discrepancy may occur normally without varicocele.

There are no clear guidelines established for treatment of a varicocele. Most varicoceles in adolescents are managed conservatively with observation. Surgical ligation of the spermatic vein, however, is usually indicated for adolescents who demonstrate retarded growth of the left testis and in young men who develop testicular atrophy [1-3]. The discrepancy of testicular volumes is the main criterion for performing surgery and may be assessed by ultrasound [4]. At times there is no asymmetry and the levels of FSH and LH may be helpful to identify patients who need surgical treatment [5]. One can easily see, looking at the table, the volume change expected from the difference of 1, 2, or 3, mm in the width. A difference of 3 mm in the width should easily be detected by the same observer (i.e. 2.0 cm to 2.3 or 2.3 to 2.6).

**Table 6:** Testicular Volumes (ml)

**Width of testis (cm)** **(ss subtracted)**	**Formulas equivalent to the following US Equations**		
	**W^2^ x L x 0.52**	**W x H x L x 0.52**	**W x H x L x 0.71**
	**(W-ss)^3^ x 0.8**	**(W-ss)^3^ x 0.64**	**(W-ss)^3^ x 0.88**
1.0	0.80	0.64	0.88
1.1	1.06	0.85	1.17
1.2	1.38	1.11	1.52
1.3	1.76	1.41	1.93
1.4	2.20	1.76	2.41
1.5	2.70	2.16	2.97
1.6	3.28	2.62	3.60
1.7	3.93	3.14	4.32
1.8	4.67	3.73	5.13
1.9	5.49	4.39	6.04
2.0	6.40	5.12	7.04
2.1	7.41	5.93	8.15
2.2	8.52	6.81	9.37
2.3	9.73	7.79	10.71
2.4	11.06	8.85	12.17
2.5	12.50	10.00	13.75
2.6	14.06	11.25	15.47
2.7	15.75	12.60	17.32
2.8	17.56	14.05	19.32
2.9	19.51	15.61	21.46
3.0	21.60	17.28	23.76
3.1	23.83	19.07	26.22
3.2	26.21	20.97	28.84
3.3	28.75	23.00	31.62

## Appendix B. References

 1. Costabile RA, Skoog S, Radowich M. **Testicular volume assessment in the adolescent with a varicocele.***J Urol*. 1992, **147**(5):1348-50.

 2. Paduch DA, Niedzielski J. **Repair versus observation in adolescent varicocele: a prospective study.***J Urol*. 1997, **158**(3 Pt 2):1128-32.

 3. Sayfan J, Siplovich L, Koltun L, Benyamin N. **Varicocele treatment in pubertal boys prevents testicular growth arrest.***J Urol*. 1997, **157**(4):1456-7.

 4. Diamond DA, Paltiel HJ, DiCanzio J, Zurakowski D, Bauer SB, Atala A, Ephraim PL, Grant R, Retik AB. **Comparative assessment of pediatric testicular volume: orchidometer versus ultrasound**. *J Urol*. 2000, 164(3 Pt 2):1111-4.

 5. Guarino N, Tadini B, Bianchi M. **The adolescent varicocele: the crucial role of hormonal tests in selecting patients with testicular dysfunction.***J Pediatr Surg*. 2003, **38**(1):120-3.

## Competing interests

The authors declare that they have no competing interests.

## Authors’ contributions

JFS contributed to conception and design, acquisition of data, analysis and interpretation of data. NJT contributed to collection, analysis and presentation of data. Both contributed to the drafting of the manuscript and the final version.
